# Gender difference in association between H-type hypertension and subcortical ischemic vascular disease

**DOI:** 10.3389/fnagi.2022.998268

**Published:** 2022-09-29

**Authors:** Juan Wang, Yuan-Xue Xi, Jia-Qi Li, Wei-Wen Zhu

**Affiliations:** ^1^The First Affiliated Hospital of Guangzhou Medical University, Guangzhou, China; ^2^School of Public Health, Guangzhou Medical University, Guangzhou, China; ^3^Department of Neurology, The Second Affiliated Hospital of Guangzhou Medical University, Guangzhou, China

**Keywords:** H-type hypertension, subcortical ischemic vascular disease, gender, cognitive function, homocysteine

## Abstract

**Background:**

Subcortical ischemic vascular disease (SIVD) is a leading cause of vascular dementia. The present study tries to explore not only the gender-specific association between H-type hypertension and SIVD but also the indirect effects of H-type hypertension on cognition through the ischemic brain injury caused by SIVD.

**Materials and methods:**

A total of 601 SIVD patients were included, comprising 322 males and 279 females. H-type hypertension was defined as hypertension accompanied with elevated serum total homocysteine (tHcy) level. The imaging manifestations of ischemic brain injury caused by SIVD were also evaluated, including white matter lesions (WML), lacunar infarction (LI) and brain atrophy (BA). Gender-specific subgroup analyses in association between H-type hypertension and SIVD were conducted, followed by a structural equation model based evaluation of the gender-specific mediating effects of SIVD on the relationship between H-type hypertension and cognition.

**Results:**

For males, there was no noticeable difference in WML, LI and BA scores among control group, isolated hypertension group, isolated high tHcy group, and H-type hypertension group in most brain regions, but significant difference was found in all brain regions for females. Multiple regression analyses showed that H-type hypertension was significantly associated with WML, LI and BA for females, but not for males. For males, H-type hypertension mainly affected cognition through direct effect, while the H-type hypertension effect was mediated by ischemic brain injury caused by SIVD for females.

**Conclusion:**

H-type hypertension was more closely related to SIVD for females than males, suggesting a gender-specific difference in association patterns between H-type hypertension and cognition.

## Introduction

Subcortical ischemic vascular disease (SIVD) is the most prevalent type of cerebral small vessel disease and a leading cause of vascular dementia (Roman et al., 2002; METACOHORTS Consortium, 2016; Ter Telgte et al., 2018). The main imaging manifestations of ischemic brain injury caused by SIVD are white matter lesions (WML), lacunar infarction (LI) and brain atrophy (BA), which are very common among people over 60 years old (Roman et al., 2002; Cannistraro et al., 2019). SIVD is closely intertwined with cognitive decline and depressive disorders caused by cerebral vascular injury, which is now referred as “vascular cognitive impairment” (VCI) and “vascular depression” (Prins et al., 2005; Cantone et al., 2020). Furthermore, CT or MRI evidence of SIVD may also be found in patients with no neurological history in the diagnosis of other disorders, referred to as “covert” cerebral small vessel disease (ccSVD). (Liu et al., 2021; Wardlaw et al., 2021). However, as the disease progresses, VCI-no dementia or vascular dementia in SIVD leads to gait disturbances, functional disability and a severe reduction in quality of life, ultimately a higher risk of death (Kim et al., 2016; Kang et al., 2022). SIVD imposes a huge financial burden on families and societies (The Lancet Neurology, 2018).

A large number of studies suggest that the prognosis of patients with acute or chronic cerebrovascular diseases is closely related to hypertension (Chow et al., 2013; Falaschetti et al., 2014; Cantone et al., 2021). Serum total homocysteine (tHcy) is a natural sulfur-containing amino acid produced during methionine metabolism pathway (Spence, 2007), and has been implicated in the pathogenesis of SIVD (Hooshmand et al., 2016; Staszewski et al., 2018; Nam et al., 2019). H-type hypertension is defined as hypertension accompanied with elevated tHcy level (Li et al., 2015). In China, 50.2% of the elderly over 60 years old suffer from H-type hypertension (Ma et al., 2017). Researchers found that H-type hypertension accounts for up to 75% of patients with essential hypertension (Kong et al., 2021; Tu et al., 2021), and increases the risk of cerebrovascular diseases (Li et al., 2015, 2020; Kong et al., 2021). However, few research has explored the relationship between H-type hypertension and SIVD, and it has remained unclear whether or not there is a gender difference on this association. Previous researches had shown that gender not only affects the prevalence of hypertension and tHcy level (Doumas et al., 2013; Huang et al., 2021), but also influences their relationships with cardiovascular and cerebrovascular diseases (Wang et al., 2015; Zhong et al., 2017; Hester et al., 2019; Madsen et al., 2019). Madsen et al found that the risk of stroke is higher in women than men with hypertension (Madsen et al., 2019). Study had reported that females are more likely to develop pulmonary hypertension than males, which is related to “Estrogen paradox” (Hester et al., 2019). Furthermore, the management of hypertension in patients with cerebrovascular disease remains controversial and outcome may vary by sex (Cantone et al., 2021). In addition, plasma tHcy level had been identified as an independent predictor of risk and outcome of stroke for women with acute ischemic stroke, but not for men (Wang et al., 2015; Zhong et al., 2017). Therefore, there may be a gender-specific correlation between H-type hypertension and SIVD.

Cognitive impairment is a core clinical feature of SIVD, resulting in a huge disease burden (METACOHORTS Consortium, 2016). Recent studies have confirmed hypertension and high tHcy concentration as risk factors for cognitive function (Mills et al., 2016; Smith et al., 2018; Nelson et al., 2021). However, to the best of our knowledge, few study has been carried out on the association of H-type hypertension and cognitive function in SIVD population. The potential mechanism remains unclear. Prior studies had suggested that cognition is independently associated with MRI features of SIVD such as WML, LI, and BA (Makin et al., 2013; Zamboni et al., 2017; Chen et al., 2021). The question is whether H-type hypertension is a direct cause of cognitive impairment in SIVD, or it indirectly affects the cognitive function through these ischemic brain injury caused by SIVD? Moreover, whether or not gender moderates the detrimental effect of H-type hypertension on cognitive function through SIVD has yet to be explored.

The aim of this study is to investigate gender differences in association between H-type hypertension and SIVD, and explore whether SIVD mediates the relationship between H-type hypertension and cognitive function, as well as related gender differences.

## Materials and methods

### Study population

A total of 601 SIVD patients who were consecutively admitted to the Department of Neurology, Second Affiliated Hospital of Guangzhou Medical University between June 2018 and April 2022 were eligible for inclusion. All patients met the brain imaging criteria proposed by Erkinjuntti T (Roman et al., 2002), and were referred to recent and clearly defined radiological criteria: (1) WML were detected as hyperintensities 5mm or larger in diameter on T2-weighted or FLAIR MRI scans (Ederle et al., 2013, Kim et al., 2014). Ratings were completed by two experienced neurological physicians according to age-related white matter change scale (ARWMC) (Wahlund et al., 2001) generated for the following five regions on each hemisphere: frontal lobe, parieto-occipital lobe, temporal lobe, infratentorial area (including brain stem and cerebellum), and basal ganglia area (0 = absence of WMLs; 1 = punctate WMLs; 2 = early confluent WMLs; 3 = confluent WML). ARWMC scores of different brain regions were the sum of scores of the left and right hemispheres. The total ARWMC scores were then calculated and ranged from 0 to 30 points; (2) LI was defined as focal hyperintensities of 3∼15 mm in the subcortical white matter or deep gray matter on T2-weighted images (Wardlaw et al., 2013; Kang et al., 2022). In order to distinguish LI from the WMLs, the hyperintensities in white matter region also required hypointensities on both T1-weighted images and diffusion-weighted imaging (Kang et al., 2022). The lesion numbers of LI was counted from the five brain regions used in the WML evaluation mentioned above. The number of LI in each brain region was the sum of those of the left and right hemispheres; (3) T1-weighted images were used to explore regional BA according to a 4-point visual rating scale (0 = no atrophy; 1 = mild atrophy; 2 = moderate atrophy; 3 = severe atrophy) (Fumagalli et al., 2018). BA score of each brain region was the sum of those of the left and right hemispheres. The overall score for five regions on each hemisphere were then calculated and ranged from 0 to 30 points. The Cronbach’s alpha value of the SIVD MRI characteristics scale was 0.712.

The following exclusion criteria were then applied: history of brain tumor, cerebral hemorrhage and brain trauma, presence of severe unrelated central nervous disease (e.g., Parkinson’s disease, Alzheimer’s disease, Lewy dementia and other degenerative diseases) and leukoencephalopathy of non-vascular origin, presence of physical reasons for failure to complete the questionnaire (e.g., Hearing, vision and language disorders). The average age of these patients was 73.49 ± 8.60 years old, and there were more males (53.5%) than females (46.5%).

### Clinical data collection

Demographic information were collected from these patients, including age, gender, educational level, history of smoking and alcohol intake, history of hypertension, hypertension duration, hypertension drug taken, history of hyperhomocysteinemia, and history of coronary heart disease. Venous blood samples were collected from SIVD patients to detect the levels of plasma tHcy, glycosylated hemoglobin (HbA1c), total cholesterol (TC), triglyceride (TG), high density lipoprotein (HDL-C) and low density lipoprotein (LDL-C).

Hypertension was defined as seated, resting systolic blood pressure (SBP) ≥140 mmHg or diastolic blood pressure (DBP) ≥90 mmHg or self-reported history of hypertension. High tHcy is defined as tHcy concentration ≥10μmol/L (Li J. et al., 2016). H-type hypertension is characterized by the combination of hypertension and high level of Hcy (Li et al., 2015), and measured on a four-point scale (0 = control; 1 = isolated hypertension; 2 = isolated high tHcy; 3 = H-type hypertension).

### Neuropsychological examination

Neuropsychological examination was performed by an experienced neurological physician using the mini-mental state examination (MMSE). MMSE consisted of 5 items in its evaluation of orientation (10 points), memory registration (3 points), attention and calculation (5 points), recall (3 points) and language (9 points) (Li H. et al., 2016). Total scores ranged from 0 to 30 points, with higher scores indicating better cognitive status.

### Statistical analysis

**FIGURE 1:**
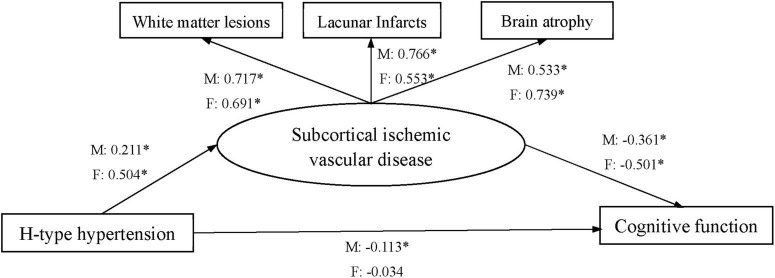
Structural equation model of the relationship between H-type hypertension, subcortical ischemic vascular disease, and cognitive function. M: male, F: female. All factor loadings were standardized; *P < 0.05.

Data analyses were performed using the SPSS 25.0 and AMOS 21.0 software packages. Numerical variables were presented as the mean and standard deviations. Comparison between groups for numerical variables was carried out with ANOVA. *Post hoc* analyses were performed via the Student-Newman-Keuls multiple comparison test. Categorical variables were summarized as frequency and percentage and analyzed using the Chi-square test. Gender-specific subgroup analyses in association between H-type hypertension and SIVD were conducted. Multiple linear regression analyses were performed to study the gender-specific relationships between H-type hypertension, WML, LI, BA and cognitive function adjusted for demographics and the plasma biochemical parameters. Structural equation modeling (SEM) technique was employed to evaluate the gender-specific mediating effects of WML, LI, and BA on the relationship between H-type hypertension and cognitive function (Preacher and Hayes, 2008). The model structure was shown in Figure 1. The goodness of fit was evaluated using the following indices: chi-square/df ratio, comparative fit index (CFI > 0.90), and Tucker-Lewis index (TLI > 0.90), root mean square of approximation (RMSEA < 0.08).

## Results

### Demographics and the plasma biochemical parameters

The demographic characteristics and the plasma biochemical parameters were presented in Table 1. Male SIVD patients had higher education, and were significantly more likely to report smoking and drinking than female patients. The female SIVD patients showed lower tHcy level and prevalence of H-type hypertension but higher TC, TG, HDL-C and LDL-C than their male counterparts.

**TABLE 1:** Demographic characteristics and the plasma biochemical parameters by gender.

	All *n* = 601	Male *n* = 322 (53.5%)	Female *n* = 279 (46.5%)	*P-value*
Age, years	73.49 ± 8.60	72.92 ± 8.62	74.14 ± 8.55	0.085
Education				<0.001
Illiteracy	133(22.1)	46(14.3)	87(31.2)	
Primary school	189(31.4)	93(28.9)	96(34.4)	
Middle school or above	279(46.4)	183(56.8)	96(34.4)	
Smoking	133(22.1)	127(39.4)	6(2.2)	<0.001
Drinking	62(10.3)	57(17.7)	5(1.8)	<0.001
History of hypertension	403 (67.1)	214 (66.5)	189 (67.7)	0.739
Hypertension duration, year				0.082
None	198 (32.9)	106 (32.9)	92 (33.0)	
0–5	155 (25.8)	94 (29.2)	61 (21.9)	
6–10	132 (22.0)	70 (21.7)	62 (22.2)	
>10	116 (19.3)	52 (16.1)	64 (22.9)	
Hypertension drug taken	307 (76.2)	155 (72.4)	152 (80.4)	0.060
History of hyperhomocysteinemia	21 (3.5)	14 (4.3)	7 (2.5)	0.221
History of coronary heart disease	64 (10.6)	31 (9.6)	33 (11.8)	0.383
SBP, mmHg	145.99 ± 22.07	146.78 ± 21.55	145.08 ± 22.63	0.357
DBP, mmHg	83.60 ± 12.39	84.41 ± 12.05	82.68 ± 12.72	0.093
tHcy, mmol/L	12.62 ± 5.28	13.63 ± 5.30	11.46 ± 5.02	<0.001
HbA1c, %	6.34 ± 1.52	6.25 ± 1.42	6.43 ± 1.62	0.141
TC, mmol/L	4.47 ± 1.17	4.21 ± 1.04	4.76 ± 1.24	<0.001
TG, mmol/L	1.46 ± 0.84	1.36 ± 0.78	1.57 ± 0.88	0.002
HDL-C, mmol/L	1.14 ± 0.32	1.08 ± 0.31	1.19 ± 0.32	<0.001
LDL-C, mmol/L	2.86 ± 1.12	2.70 ± 1.05	3.03 ± 1.17	<0.001
H-type hypertension				<0.001
Control	45 (7.5)	13 (4.0)	32 (11.5)	
Isolated hypertension	143 (23.8)	47 (14.6)	96 (34.4)	
Isolated High tHcy	60 (10.0)	39 (12.1)	21 (7.5)	
H-type hypertension	353 (58.7)	223 (69.3)	130 (46.6)	

Numerical variables are expressed as mean ± standard deviation, categorical variables are expressed as frequency (percent). SBP, systolic blood pressure; DBP, diastolic blood pressure; HbA1c, glycosylated hemoglobin A1c; TC, total cholesterol; TG, triglycerides; HDL-C, high-density lipoprotein cholesterol; LDL-C, low-density lipoprotein cholesterol.

### White matter lesions, lacunar infarction, brain atrophy and cognitive function

The scores of WML, LI, and BA for frontal lobe, parietal-occipital lobe, temporal lobe, infratentorial area, and basal ganglia area among male SIVD patients were shown in Table 2, which showed higher WML scores in the parieto-occipital lobe, basal ganglia area, and total brain regions, BA scores in the frontal lobe, temporal lobe for the H-type hypertension group than those in both the isolated hypertension group and the isolated high tHcy group. There was a significant difference in WML scores of the frontal lobe between the isolated high tHcy group and the H-type hypertension group. Higher BA scores in the basal ganglia area was observed in the H-type hypertension group when compared against those in the isolated hypertension group.

**TABLE 2:** White matter lesions, lacunar infarction, brain atrophy and cognitive function according to H-type hypertension for males.

Variables	Male					
	All *n* = 322	Control *n* = 13 (4.0%)	Isolated hypertension *n* = 47 (14.6%)	Isolated high tHcy *n* = 39 (12.1%)	H-type hypertension *n* = 223 (69.3%)	*P-value*
**WML**						
Frontal lobe	3.63 ± 1.77	3.03 ± 2.30	3.32 ± 1.46	2.88 ± 1.35	3.86 ± 1.81^c^	0.003
Parieto-occipital lobe	3.22 ± 1.94	2.84 ± 2.17	2.72 ± 1.64	2.66 ± 1.53	3.44 ± 2.02^b^^c^	0.021
Temporal lobe	0.75 ± 1.18	0.34 ± 0.85	0.62 ± 1.11	0.56 ± 0.96	0.83 ± 1.24	0.242
Infratentorial area	0.67 ± 1.09	0.53 ± 1.33	0.60 ± 0.80	0.57 ± 0.92	0.72 ± 1.16	0.785
Basal ganglia area	2.34 ± 1.75	1.80 ± 2.06	1.87 ± 1.54	1.94 ± 1.39	2.54 ± 1.80^b^^c^	0.021
Total score	10.63 ± 5.89	8.57 ± 7.01	9.15 ± 4.93	8.64 ± 4.19	11.41 ± 6.11^b^^c^	0.004
**LI**						
Frontal lobe	1.35 ± 1.63	1.00 ± 1.47	1.15 ± 1.58	0.87 ± 1.05	1.50 ± 1.71	0.091
Parieto-occipital lobe	0.54 ± 1.17	0.38 ± 0.96	0.46 ± 1.06	0.30 ± 0.69	0.61 ± 1.27	0.418
Temporal lobe	0.62 ± 1.21	0.73 ± 1.05	0.40 ± 0.83	0.48 ± 1.07	0.68 ± 1.30	0.428
Infratentorial area	0.82 ± 1.41	0.15 ± 0.55	0.91 ± 1.51	0.79 ± 1.19	0.85 ± 1.44	0.352
Basal ganglia area	3.53 ± 2.79	2.30 ± 2.95	3.25 ± 2.98	3.05 ± 2.70	3.74 ± 2.75	0.144
Total score	6.88 ± 5.64	4.57 ± 5.19	6.20 ± 5.84	5.51 ± 4.33	7.40 ± 5.77	0.069
**BA**						
Frontal lobe	2.85 ± 1.59	2.61 ± 2.06	2.42 ± 1.52	2.48 ± 1.39	3.02 ± 1.59^b^^c^	0.038
Parieto-occipital lobe	2.64 ± 1.57	2.46 ± 2.11	2.36 ± 1.79	2.52 ± 1.60	2.73 ± 1.49	0.443
Temporal lobe	3.76 ± 1.66	3.15 ± 2.33	3.30 ± 1.71	3.35 ± 1.76	3.96 ± 1.57^b^^c^	0.012
Infratentorial area	1.84 ± 1.66	1.69 ± 1.97	1.70 ± 1.54	1.79 ± 1.55	1.88 ± 1.70	0.893
Basal ganglia area	3.20 ± 1.60	2.61 ± 2.21	2.74 ± 1.58	2.87 ± 1.59	3.38 ± 1.55^b^	0.017
Total score	14.30 ± 7.03	12.53 ± 9.92	12.54 ± 7.14	13.04 ± 6.93	15.00 ± 6.77	0.064
**MMSE**						
Orientation	8.56 ± 2.26	9.46 ± 1.39	9.21 ± 1.57	8.58 ± 1.98	8.36 ± 2.44	0.054
Memory registration	2.64 ± 0.78	2.76 ± 0.43	2.80 ± 0.61	2.64 ± 0.84	2.59 ± 0.81	0.339
Attention and calculation	3.15 ± 1.82	4.07 ± 1.80	3.53 ± 1.74	3.20 ± 1.71	3.00 ± 1.84	0.080
Recall	1.77 ± 1.11	2.46 ± 0.77	2.06 ± 1.03	1.66 ± 1.17^a^	1.69 ± 1.12^a^^b^	0.022
Language	7.38 ± 1.84	8.53 ± 0.66	7.87 ± 1.60	7.58 ± 1.25	7.17 ± 1.97^a^^b^	0.008
Total score	23.51 ± 6.29	27.30 ± 3.81	25.48 ± 5.00	23.69 ± 5.21	22.86 ± 6.67^a^^b^	0.008

^a^*P* < 0.05 vs. control group, ^b^*P* < 0.05 vs. isolated hypertension group, ^c^*P* < 0.05 vs. isolated high tHcy group. Numerical variables are expressed as mean ± standard deviation. Same alphabetic superscripts illustrate no significant difference. WML, white matter lesions; LI, lacunar infarction; BA, brain atrophy; MMSE, mini-mental state examination.

The scores of each cognitive domain and the total MMSE score for male SIVD patients were also shown in Table 2, which showed lower scores of recall, language and the total MMSE score for the H-type hypertension group when compared with those of the control group and the isolated hypertension group. There were significant difference in the scores of recall between the control group and the isolated high tHcy group.

Female SIVD patients with H-type hypertension showed higher WML scores for frontal lobe, parietal-occipital lobe, temporal lobe, and basal ganglia area, higher LI score for frontal lobe, infratentorial area, basal ganglia area, and higher BA scores for all brain regions, when compared with those in the isolated hypertension group and the control group (Table 3). Higher WML scores in both the temporal lobe and infratentorial area as well as higher LI scores in the frontal lobe were observed in the isolated high tHcy group when compared against those in the isolated hypertension and the control group. Between the isolated high tHcy group and the control group, there were significant differences in WML score in the frontal lobe, LI score in the basal ganglia area, and BA scores for all brain regions. Similarly, in comparison to the control group, the isolated hypertension group got higher WML score in the frontal lobe, higher LI score in the frontal lobe, and higher BA score in the frontal lobe, parietal-occipital lobe, temporal lobe, and basal ganglia area.

**TABLE 3:** White matter lesions, lacunar infarction, brain atrophy and cognitive function according to H-type hypertension for females.

Variables	Female					
	All *n* = 279	Control *n* = 32 (11.4%)	Isolated hypertension *n* = 96 (34.5%)	Isolated high tHcy *n* = 21 (7.5%)	H-type hypertension *n* = 130 (46.6%)	*P-value*
**WML**						
Frontal lobe	3.51 ± 1.82	2.42 ± 1.58	3.15 ± 2.00^a^	3.76 ± 1.47^a^	4.01 ± 1.61^a^^b^	<0.001
Parieto-occipital lobe	2.79 ± 1.95	1.79 ± 1.50	2.46 ± 2.01	2.81 ± 1.88	3.27 ± 1.90^a^^b^	<0.001
Temporal lobe	0.45 ± 0.96	0.01 ± 0.08	0.24 ± 0.67	0.76 ± 1.10^a^^b^	0.67 ± 1.14^a^^b^	<0.001
Infratentorial area	0.57 ± 1.09	0.35 ± 0.91	0.30 ± 0.73	1.00 ± 1.47^a^^b^	0.75 ± 1.23^b^	0.003
Basal ganglia area	2.29 ± 1.75	1.81 ± 1.54	1.88 ± 1.70	2.45 ± 1.65	2.69 ± 1.77^a^^b^	0.002
Total score	9.63 ± 5.82	6.40 ± 4.33	8.05 ± 5.64	10.78 ± 5.52^a^^b^	11.41 ± 5.71^a^^b^	<0.001
LI						
Frontal lobe	1.18 ± 1.55	0.31 ± 0.73	0.93 ± 1.26^a^	1.66 ± 1.59^a^^b^	1.50 ± 1.77^a^^b^	<0.001
Parieto-occipital lobe	0.24 ± 0.75	0.09 ± 0.53	0.15 ± 0.60	0.28 ± 0.64	0.33 ± 0.89	0.224
Temporal lobe	0.39 ± 0.93	0.06 ± 0.24	0.32 ± 0.81	0.33 ± 0.65	0.57 ± 1.05^a^	0.044
Infratentorial area	0.40 ± 0.90	0.21 ± 0.79	0.18 ± 0.48	0.61 ± 1.24^b^	0.71 ± 1.15^a^^b^	0.005
Basal ganglia area	2.74 ± 1.90	1.93 ± 1.50	2.47 ± 1.95	3.28 ± 1.82^a^	3.05 ± 1.89^a^^b^	0.004
Total score	4.97 ± 3.83	2.62 ± 2.10	4.08 ± 3.29	6.19 ± 3.07^a^^b^	6.01 ± 4.22^a^^b^	<0.001
**BA**						
Frontal lobe	2.55 ± 1.69	1.21 ± 1.23	2.31 ± 1.76^a^	2.64 ± 1.62^a^	3.05 ± 1.55^a^^b^	<0.001
Parieto-occipital lobe	2.29 ± 1.66	0.98 ± 1.27	2.03 ± 1.65^a^	2.52 ± 1.81^a^	2.78 ± 1.53^a^^b^	<0.001
Temporal lobe	3.18 ± 1.79	1.87 ± 1.64	2.77 ± 1.73^a^	3.50 ± 1.79^a^	3.76 ± 1.63^a^^b^	<0.001
Infratentorial area	1.27 ± 1.45	0.40 ± 0.91	0.94 ± 1.32	1.33 ± 1.39^a^	1.71 ± 1.52^a^^b^	<0.001
Basal ganglia area	2.41 ± 1.72	1.34 ± 1.61	2.04 ± 1.72^a^	2.57 ± 1.88^a^	2.93 ± 1.55^a^^b^	<0.001
Total score	11.72 ± 7.21	5.82 ± 5.85	10.11 ± 7.14^a^	12.57 ± 6.97^a^	14.23 ± 6.46^a^^b^	<0.001
**MMSE**						
Orientation	8.19 ± 2.55	9.53 ± 0.94	8.67 ± 2.14	7.57 ± 2.90^a^	7.61 ± 2.85^a^^b^	<0.001
Memory registration	2.65 ± 0.72	2.75 ± 0.72	2.73 ± 0.57	2.85 ± 0.35	2.53 ± 0.84	0.086
Attention and calculation	2.74 ± 1.86	3.50 ± 1.72	3.23 ± 1.67	2.61 ± 1.96	2.20 ± 1.87^a^^b^	<0.001
Recall	1.75 ± 1.11	2.15 ± 0.88	1.95 ± 1.03	1.71 ± 1.23	1.50 ± 1.14^a^^b^	0.003
Language	7.13 ± 1.96	7.75 ± 1.43	7.55 ± 1.67	6.42 ± 2.01^a^^b^	6.79 ± 2.15^a^^b^	0.002
Total score	22.47 ± 6.50	25.68 ± 4.26	24.14 ± 5.12	21.19 ± 6.22^a^^b^	20.66 ± 7.29^a^^b^	<0.001

^a^*P* < 0.05 vs. control group, ^b^*P* < 0.05 vs. isolated hypertension group, ^c^*P* < 0.05 vs. isolated high tHcy group. Numerical variables are expressed as mean ± standard deviation. Same alphabetic superscripts illustrate no significant difference. WML, white matter lesions; LI, lacunar infarction; BA, brain atrophy; MMSE, mini-mental state examination.

The scores of each cognitive domain and the total MMSE score for female SIVD patients were also shown in Table 3. Lower scores of orientation, attention and calculation, recall, language and a lower total MMSE score were observed in the H-type hypertension group when compared against those in the control group and the isolated hypertension group. Female SIVD patients with isolated high tHcy level had lower language and total MMSE score than those in the isolated hypertension group and the control group.

### Multiple linear regressions between total homocysteine, age-related white matter change scale, lacunar infarction, brain atrophy, and mini-mental state examination

Among all SIVD patients, H-type hypertension was found positively associated with WML, LI, and BA, but negatively associated with MMSE in multiple regression model adjusted for age, education, smoking, drinking, history of coronary heart disease, HbA1c, TC, TG, HDL-C and LDL-C (Table 4).

**TABLE 4:** Multiple linear regression between H-type hypertension and age-related white matter change scale, lacunar infarction, brain atrophy, mini-mental state examination.

Dependent variables	Independent variable		

	Total		
	Isolated hypertension	Isolated high tHcy	H-type hypertension
WML	0.801 (–1.209, 2.811)	1.498 (–0.817, 3.814)	3.692 (1.759, 5.624)**
LI	1.282 (–0.454, 3.018)	1.916 (–0.084, 3.915)	3.091 (1.422, 4.760)**
BA	1.093 (–1.119,3.305)	1.989 (–0.559, 4.537)	3.394 (1.268, 5.521)*
MMSE	–0.485 (–2.618, 1.648)	–2.087 (–4.543, 0.370)	–2.626 (–4.676, -0.575)*

	**Male**		
	**Isolated hypertension**	**Isolated high tHcy**	**H-type hypertension**

WML	–0.637 (–4.698, 3.425)	–0.857 (–4.932, 3.218)	1.880 (–1.890, 5.649)
LI	0.870 (–3.124, 4.863)	0.144 (–3.862, 4.151)	1.941 (–1.765, 5.647)
BA	–1.453 (–5.810, 2.904)	–1.608 (–5.979, 2.764)	–0.118 (–4.162, 3.926)
MMSE	–0.151 (–4.401, 4.099)	–1.711 (–5.974, 2.553)	–2.437 (–6.381, 1.508)

	**Female**		
	**Isolated hypertension**	**Isolated high tHcy**	**H-type hypertension**

WML	0.929 (–1.376, 3.233)	3.140 (–0.043, 6.324)	3.931 (1.567, 6.294)*
LI	1.186 (–0.346, 2.717)	3.536 (1.421, 5.652)*	3.041 (1.471, 4.611)**
BA	1.873 (–0.678, 4.424)	3.468 (–0.057, 6.992)	4.343 (1.726,6.959)*
MMSE	–0.799 (–3.289, 1.692)	–2.788 (–6.228,0.652)	–3.325 (–5.878, -0.771)*

Data are reported as Beta (95%CI). Adjusted for age, education, smoking, drinking, history of coronary heart disease, HbA1c, TC, TG, HDL-C and LDL-C. **P < 0.001. *P < 0.05.

For male, insignificant association was found between H-type hypertension and WML, LI, BA and MMSE. For female, H-type hypertension had positive associations with WML, LI, and BA, and negative association with MMSE in adjusted multiple linear regression models. In addition, significant associations between isolated high tHcy and LI were also found.

### Correlation analyses

The intercorrelations of SBP, DBP, tHcy, WML, LI, BA, and MMSE were presented in Table 5. SBP was positively correlated with WML and LI, and DBP was also positively correlated with WML in all SIVD patients. Positive associations were found between SBP and BA, as well as DBP and WML in female SIVD patients. tHcy was positively correlated with WML, LI, and BA, and negatively correlated with MMSE for all SIVD patients. Moreover, tHcy was found correlated with BA and MMSE in male SIVD patients, and significant associations between tHcy and WML, LI, BA, and MMSE were observed in female SIVD patients. SBP and DBP were significantly correlated, and WML, LI, BA, and MMSE were intercorrelated.

**TABLE 5:** Intercorrelations of the manifest variables used in this study.

			Total				
Variables	SBP	DBP	Hcy	WML	LI	BA	MMSE
SBP	1						
DBP	0.682**	1					
Hcy	0.053	–0.038	1				
WML	0.092*	0.090*	0.149**	1			
LI	0.087*	0.062	0.157**	0.572**	1		
BA	0.079	–0.003	0.281**	0.451**	0.403**		
MMSE	–0.012	0.059	–0.236**	–0.282**	–0.322**	–0.301**	1

			**Male**				
**Variables**	**SBP**	**DBP**	**Hcy**	**WML**	**LI**	**BA**	**MMSE**

SBP	1						
DBP	0.654**	1					
Hcy	0.029	–0.095	1				
WML	0.095	0.037	0.086	1			
LI	0.061	0.014	0.100	0.559**	1		
BA	0.016	–0.087	0.239**	0.382**	0.389**		
MMSE	–0.069	0.014	–0.328**	–0.224**	–0.305**	–0.276**	1

			**Female**				
**Variables**	**SBP**	**DBP**	**Hcy**	**WML**	**LI**	**BA**	**MMSE**

SBP	1						
DBP	0.709**	1					
Hcy	0.065	–0.007	1				
WML	0.082	0.138*	0.196**	1			
LI	0.118	0.109	0.166**	0.609**	1		
BA	0.133*	0.057	0.272**	0.516**	0.391**		
MMSE	0.041	0.095	–0.178**	–0.368**	–0.427**	–0.370**	1

**P < 0.001. *P < 0.05.

### Structural equation model

The structural equation model that included the mediator (SIVD) for all SIVD patients was evaluated. The direct effect of H-type hypertension on cognitive function was insignificant for all SIVD patients (Table 6). The indirect effect in the mediated model with a 95% confidence interval were shown in Table 6, which suggested that an increase in H-type hypertension Level by 1 standard deviation (SD) was significantly associated with a decrease in level of cognitive function by 0.147 SD through SIVD. The relevant indices showed that the model has good fitting effect: χ^2^/df = 4.202, CFI = 0.977, TLI = 0.943, RMSEA = 0.073.

**TABLE 6:** Direct and indirect effects for the mediation analyses.

Pathway	Total		
Direct effect	Estimated effect	Critical ratio	*P-value*
H-type hypertension→SIVD	0.369	7.962	<0.001
SIVD→cognitive function	–0.397	–7.457	<0.001
H-type hypertension→cognitive function	–0.065	–1.541	0.123

**Indirect effect**	**Estimated effect**	**95%CI**	***P-value***

H-type hypertension→SIVD→cognitive function	–0.147	(–0.194, –0.101)	0.012

	**Male**		
**Direct effect**	**Estimated effect**	**Critical ratio**	***P-value***

H-type hypertension→SIVD	0.211	3.184	0.001
SIVD→cognitive function	–0.361	–5.167	<0.001
H-type hypertension→cognitive function	–0.113	–2.075	0.038

**Indirect effect**	**Estimated effect**	**95%CI**	***P-value***

H-type hypertension→SIVD→cognitive function	–0.076	(–0.116, –0.034)	0.008

	**Female**		
**Direct effect**	**Estimated effect**	**Critical ratio**	***P-value***

H-type hypertension→SIVD	0.504	6.824	<0.001
SIVD→cognitive function	–0.501	–5.450	<0.001
H-type hypertension→cognitive function	–0.034	–0.507	0.612

**Indirect effect**	**Estimated effect**	**95%CI**	***P-value***

H-type hypertension→SIVD→cognitive function	–0.252	(–0.340, –0.168)	0.011

The gender-specific mediating effects of WML, LI, and BA on the relationship between H-type hypertension and cognitive function were also examined. The direct standardized path coefficients were shown, respectively, for male (M) and female (F) in Figure 1. The direct effects of H-type hypertension on cognitive function were significant for males, but insignificant for females. The direct standardized path coefficient indicated that an increase in H-type hypertension level by 1 SD was significantly associated with a decrease in level of cognitive function by 0.113 SD for male SIVD patients. The indirect effects indicated that an increase in H-type hypertension Level by 1 SD was significantly associated with a decrease in level of cognitive function by 0.076/0.252 SD through SIVD for male and female SIVD patients. The following indices showed that the model fit the data well: χ^2^/df = 1.879/1.086, CFI = 0.985/0.999, TLI = 0.963/0.997, RMSEA = 0.052/0.018.

## Discussion

Our analyses showed a gender difference in association between H-type hypertension and SIVD. H-type hypertension was more closely related to SIVD for females than for males. In addition, we found difference in the association patterns between H-type hypertension and cognition for SIVD patients of different genders.

This study found that, despite the proportion of male patients with H-type hypertension was higher than female patients with H-type hypertension, H-type hypertension brought a higher risk of ischemic brain injury in most brain regions to female SIVD patients, but it was not obvious for male. For males, there was no significant difference in WML, LI and BA scores among the control group, the isolated hypertension group, the isolated high tHcy group, and the H-type hypertension group, except for the scores of WML in frontal lobe, parietal-occipital lobe, basal ganglia area and whole brain, as well as the BA scores in frontal lobe, temporal lobe, and basal ganglia area. For females, WML, LI and BA scores were significantly different among the control group, the isolated hypertension group, the isolated high tHcy group, and the H-type hypertension group in all brain regions. Multiple regression models showed that H-type hypertension was significantly associated with WML, LI and BA for females, but not for males. Previous researches had shown that the prevalence of hypertension and tHcy level were higher in males than in females (Doumas et al., 2013; Huang et al., 2021), the associations between hypertension, tHcy, cardiovascular and cerebrovascular diseases were also different between genders (Wang et al., 2015; Zhong et al., 2017). A recent study had reported that the association between the increased hypertension severity and the onset of ischemic stroke was almost twice as strong in women as in men (Madsen et al., 2019). In addition, Voigt et al reported that the total and proximal plaque burdens were significantly higher for hypertensive male patients with acute ischemic stroke than for their female counterparts (Voigt et al., 2021). Zhong et al reported that elevated plasma tHcy level predicted the poor prognosis of acute ischemic stroke for women, but not for men (Zhong et al., 2017). A recent study by Wang et al. showed that the risk of ischemic stroke associated with tHcy was more likely to be statistically significant higher for women (Wang et al., 2015). One hypothesis is that gender difference in the relationship between H-type hypertension and SIVD may due to estrogen, which has been reported to negatively regulate the prevalence of hypertension and homocysteine levels (Kuo et al., 2005; Ashraf and Vongpatanasin, 2006). Moreover, tHcy had been reported to be associated with endothelial dysfunction only in hypertensive women (Zhong et al., 2017). Most of our female subjects were postmenopausal women with reduced level of estrogen, who had been reported to be more vulnerable to endothelial dysfunction (Zuo et al., 2013). Further investigation is needed to reveal whether or not estrogen mediates an important detrimental effect in the association between H-type hypertension and SIVD.

Our results indicated that H-type hypertension, the combination of hypertension and high tHcy, presented a greater risk of SIVD than either of them alone. Hypertension has been widely proven to be associated with cerebrovascular diseases (Doumas et al., 2013; Mills et al., 2016). The effect of hypertension on SIVD and VaD may be related to the damage of blood-brain barrier, asymptomatic stroke induced vascular damage and WML, and aggravate hippocampal atrophy (Verhaaren et al., 2013; Venkat et al., 2015; Moretti and Caruso, 2020). However, the relationships between blood pressure and SIVD and VAD were still controversial, some studies suggested that low blood pressure may be more dangerous (Verghese et al., 2003; Venkat et al., 2015; Caruso et al., 2019). Study had reported that hypertension was not related to Alzheimer’s disease (Lindsay et al., 2002), and other study had found that there was no difference in the prevalence of hypertension between patients with non-lacunar and lacunar lesions, and many lacunar stroke patients had normal blood pressure (Jackson et al., 2010). Nevertheless, none of these studies have paid attention to gender differences in these associations. Elevated tHcy levels plays a decisive role in the development and progression of inflammation, atherosclerotic plaque formation, endothelial and arteriolar injury, smooth muscle cell proliferation, and oxidative stress response alterations (Moretti et al., 2021). Few study has focused on the relationship between high tHcy level and SIVD. Elevated tHcy had been thought to be involved in the pathogenesis of cerebral small vessel disease mainly by inducing reactive oxygen species production and decreasing NO production and bioavailability, triggering an increase in redox signaling and inflammation accompanying coagulation system activation, ultimately resulting in endothelial dysfunction (Pushpakumar et al., 2014; Nath et al., 2019; Thrippleton et al., 2019). Vitamins of the B group were closely associated with endothelial protection, and their deficiency was not only the most common cause of elevated homocysteine levels but also thought to be associated with SIVD pathology (Moretti and Peinkhofer, 2019). Based on multiple comparisons of the control group, the isolated hypertension group, the isolated high tHcy group, and the H-type hypertension group, we obtained more comprehensive information on the impacts of hypertension and tHcy on SIVD. For males, WML and BA were more severe in H-type hypertension group than those of the isolated tHcy and the isolated hypertension group in some brain regions. For females, H-type hypertension had a more significant impact on SIVD than isolated hypertension in most brain regions.

We also found that H-type hypertension was associated with VCI for both male and female patients with SIVD, but the pathways of association were different. VCI is considered to be the most common cognitive disorders for the elderly as it encompasses any degree of vascular-based cognitive decline (Prins et al., 2005; Wardlaw et al., 2021). SIVD is the main cause of VCI and may present with clinical manifestations of stroke, VCI-no dementia or dementia, vascular depression or physical dysfunction associated with cerebrovascular injury (Prins et al., 2005; Wardlaw et al., 2021). It is also now known as “covert” cerebral small vessel disease (ccSVD) and may be asymptomatic, discovered incidentally on neuroimaging (Wardlaw et al., 2021). For male SIVD patients, H-type hypertension mainly affected cognition through direct effect, while the indirect effect through cerebral ischemia induced by SIVD was less noticeable. However, for female SIVD patients, H-type hypertension had no direct impact on cognition, but indirectly affected cognition through cerebral ischemia injury caused by SIVD. The ischemic brain injury caused by SIVD partially mediated the effect of H-type hypertension on cognitive function for males, while completely mediated for females. Gender differences in the associations between H-type hypertension and WML, LI and BA may influence the mediating effect of cerebral ischemia injury caused by SIVD in the association between H-type hypertension and cognitive impairment. Previous study had shown that the effects of higher tHcy on cognitive function depended on the severity of white matter damage (Pavlovic et al., 2011). Brain atrophy had been shown to mediate the relationship between tHcy and cognitive decline in individuals (Mills et al., 2016). However, most studies focused solely on tHcy level and had not paid attention to the relevant gender difference. Furthermore, various SIVD imaging features often coexisted with each other, especially in the elderly (Nam et al., 2019). In the mediation analysis of this study, the latent structure of cerebral ischemia injury caused by SIVD consisted of three manifest variables in WML, LI and BA, and its mediating role in the association between H-type hypertension and cognition was analyzed by a structural equation model. The results suggested that H-type hypertension mediated subcortical ischemic brain lesions may play an important role in the pathogenesis of cognitive impairment for females, but the mechanism pathway was unclear for males. Future studies may explore the cause of gender differences in the mediating role of SIVD cerebral ischemia injury in the association between H-type hypertension and cognition, leading to more specific evidence for prevention and treatment.

To the best of our knowledge, there is no published study on gender difference in the association between H-type hypertension and SIVD. This study with a relatively large sample set suggests that female is more vulnerable than male to the deleterious effects of H-type hypertension on SIVD. However, this study had several limitations. First, due to the cross-sectional design of our study, the causal relationship between H-type hypertension and SIVD as well as its cognitive impairment remains undetermined, which needs further study to explore. Secondly, our study was a single-center study despite a relatively large sample size, a homogeneous SIVD population, and a comprehensive evaluation of all components of MRI features. Our study results could not be easily generalized to all SIVD patients. Thirdly, instead of a volumetric measurement, a visual rating scale was employed to measure WML, which is a widely used and robust measurement tool in clinical practice. Fourthly, the lack of a group of patients with H-type hypertension but no SIVD prevented a deeper analysis of the effects of gender differences. Finally, in addition to the risk factor for SIVD and the role of H-type hypertension, future study may also consider the effect of protective factors in this population, such as the role of caffeine or acetyl-L-carnitine intake on mood and cognition (Pennisi et al., 2020; Fisicaro et al., 2021).

## Conclusion

The results of this study provide valuable evidence for the prevention of the detrimental effect of H-type hypertension on SIVD and subsequent harm to cognition, and point out a new direction for both epidemic and mechanism research in related fields. The finding of gender differences in the association between H-type hypertension and SIVD was conducive to the development of precise prevention and treatment. The identification of key causes of cognitive decline was important to reduce the burden of SIVD patients and their families. Future longitudinal studies with larger sample sets are expected to verify the results of this study and find out specific mechanism.

## Data availability statement

The raw data supporting the conclusions of this article will be made available by the authors, without undue reservation.

## Ethics statement

The studies involving human participants were reviewed and approved by Ethics Committee of Guangzhou Medical University (ID: 202107013). The patients/participants provided their written informed consent to participate in this study.

## Author contributions

JW undertook the statistical analysis and wrote the first draft of the manuscript. W-WZ conceptualized and designed the study. Y-XX and J-QL made contributions to interpretation of data. All authors contributed to the article and approved the submitted version.
